# Nonlocal Conduction in a Metawire

**DOI:** 10.1002/adma.202415278

**Published:** 2025-02-21

**Authors:** Julio Andrés Iglesias Martínez, Yi Chen, Ke Wang, Martin Wegener

**Affiliations:** ^1^ Institute of Nanotechnology Karlsruhe Institute of Technology (KIT) 76128 Karlsruhe Germany; ^2^ Institute of Applied Physics Karlsruhe Institute of Technology (KIT) 76128 Karlsruhe Germany

**Keywords:** Metamaterial, Metawire, Nonlocal dc electric conduction, Nonlocal medium

## Abstract

Ohm's law of electric conduction is local in the sense that the current density at one position only depends on the electric field at that same position. For a nonlocal medium, the current density at one position depends on the electric field at other positions within the medium as well. As a result of Ohm's law, doubling the length of a wire doubles its resistance. Here, electrically conducting nonlocal architectures are discussed theoretically and experimentally for which changing the length of the metawire rather leads to a complex oscillatory behavior versus wire length. This oscillatory behavior is connected to local currents inside of the metawire flowing in the opposite direction than the externally applied field. The theoretical and experimental results for electric conduction can directly be transferred to thermal conduction or particle diffusion and may enable remote sensing applications.

## Graphical abstract

A 1D metawire composed of twisted copper wires is designed and realized. This metamaterial exhibits pronounced effects of nonlocal electric conduction according to Ohm's law. The current at one location not only depends on the electric field at that location but also on other locations. As a result, the resistance of the metawire oscillates as a function of its length.

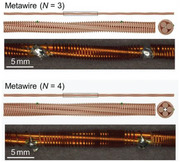

## 1 Introduction

Under many circumstances, for example in atomic metals and semiconductor crystals, electrical conduction can be described by Ohm's law.^[^ ^1^ ^]^ In its most common form, it reads *U* = *RI*, with the applied voltage *U*, the electrical current *I*, and the Ohm's resistance *R*, the inverse of the conductance *G* = 1/*R*. Ohm's law is local in the sense that, in the form ***j*** (***r***) = σ(***r***) ***E*** (***r***), with the current density ***j***, the electric field ***E***, and the electric conductivity σ, the current density at position ***r*** only depends on the electric field at that very same position ***r***. One simple and immediate measurable consequence of locality is the fact that the resistance of a wire with a fixed cross section and with length *L* is simply proportional to the wire length, i.e., 1/*G* = *R*∝*L*. One can think about doubling the length as connecting two local resistors in series, doubling the resistance.

At nonzero frequency, ω≠0, a local complex‐valued alternating current (ac) conductivity σ(ω) can be mapped onto a local dielectric function (or electric permittivity) ε(ω) via ε (ω) = ε_0_ + iσ(ω)/ω. Therefore, a nonlocal ac conductivity leads to a nonlocal dielectric function and vice versa. For ordinary metals, deviations from local behavior have extensively been discussed.^[^ ^2^, ^3^, ^4^ ^]^ Typically, for periodic atomic crystals, the characteristic length scale associated with nonlocality is on the order of one crystal lattice period *a* (on the order of half a nanometer).^[^ ^5^ ^]^ Nevertheless, highly interesting and unusual behavior can arise in nano‐plasmonics due to nonlocal extensions of Ohm's law.^[^ ^6^, ^7^, ^8^ ^]^

Here, we present rationally designed artificial periodic materials, metamaterials^[^ ^9^, ^10^, ^11^ ^]^ or “metawires”, that exhibit pronounced effects of nonlocal electric conduction in the direct current (dc) regime (zero‐frequency or static regime). For a metawire with length *L*, we find that the resistance *R*(*L*) can exhibit pronounced oscillations versus length *L*. These oscillations decay exponentially versus *L*. We show that the decay length can be tailored by the design of the metawire and that it can become much larger than the metawire period *a*, in principle even infinitely large. These oscillations of the resistance are connected to real‐space oscillations of the electric potential (at fixed wire length and fixed applied bias voltage). This means that the local electric field inside the nonlocal material is partly opposite to the externally applied electric field. As a result, contributions to the local electric currents are opposite to the mean of the current over the wire cross section. Our experiments are in excellent agreement with the theory. Nonlocal conduction might find applications in terms of remote electrical sensing.

## 2 Designing Nonlocal dc Conduction

**Figure 1:**
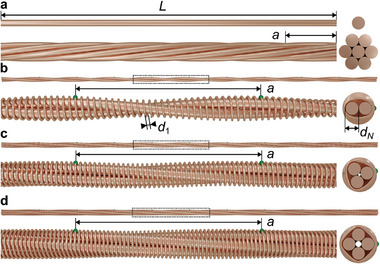
Schemes of two local a) and three nonlocal b–d) conducting wires and metawires, respectively. The period of the metawires is *a*. The integer *N* = 2, 3, 4 labelled in panels (b–d) is the order of the nonlocal interaction. The individual cylindrical wires with diameters *d*_1_ and *d_N_* are made from copper. They are all electrically isolated with respect to each other by a thin coating except for the points highlighted in green, where they are electrically connected. The insets on the right‐hand side are rendered cross sections of the wires and metawires, respectively.

**Figure** **1**a depicts two metal wires or cables used in every‐day life. These are not always straight cylinders but can be composed of helically wound wires or stranded wires. Technically, one might call such designed composite periodic architectures metamaterials or metawires, but their properties are still local and ordinary. Specifically, their Ohm's resistance *R* is simply proportional to their length *L*. Figure 1b–d exhibits three examples of nonlocal conducting metamaterial cables, the design and properties of which are the subject of the present work, which has been triggered by recent work on nonlocal static elastic behavior.^[^ ^12^ ^]^

In its simplest form, local elasticity can be described by Hooke's law. It reads *F* = *Ku*, with the applied force *F*, the displacement from equilibrium *u*, and the Hooke's spring constant *K*. When increasing the length *L* of an elastic specimen, e.g., a cylindrical beam, while maintaining its cross section, the effective spring constant monotonically decreases according to *K*(*L*)∝1/*L*. In sharp contrast, for engineered nonlocal elastic metamaterials,^[^ ^13^, ^14^, ^15^, ^16^ ^]^ we recently found pronounced oscillations of *K*(*L*) versus *L*.^[^ ^12^ ^]^ We explained these anomalous oscillations in terms of the concept of frozen evanescent phonons.^[^ ^12^ ^]^ These frozen evanescent waves are the Bloch eigenmodes of the static periodic problem. They arise from the ordinary real‐valued phonon band structure via the Cauchy‐Riemann equations applied to the complex‐valued band structure,^[^ ^17^, ^18^, ^19^, ^20^ ^]^ in which complex wavenumbers *k* represent evanescent solutions.

However, electrical conduction discussed in the present paper is a diffusion problem and not a wave problem such as phonons.^[^ ^21^ ^]^ In a wave‐type partial differential equation, the second derivative with respect to time appears, whereas in a diffusion‐type equation, only the first derivative with respect to time occurs. As a result, diffusion‐type equations do not support waves. Yet, in the static regime, all temporal derivatives are zero and we expect a close analogy between elastic behavior and electrical conduction. This analogy becomes more intuitive when considering continuum elasticity,^[^ ^22^ ^]^ in which forces *F* can be interpreted as momentum currents – to be compared with electric‐charge currents *I* in conduction. Thus, we expect closely similar behavior connected to the two equations *F* = *Ku* (Hooke's law) and *I* = *GU* (Ohm's law), respectively.

**Figure 2:**
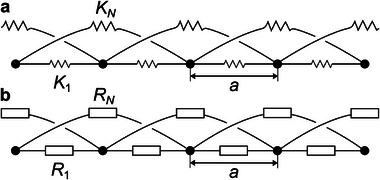
Discrete models for nonlocal metamaterials for the example of *N* = 2. a) 1D elastic metamaterial described by discrete Hooke's springs. b) Conductive metawire is described by lumped Ohm's resistors (*cf*. Figure 1b).

In the static mechanical case, masses play no role, and a simple network composed of Hooke's springs with spring constants *K*_1_ and *K*_2_ as shown in **Figure** **2**a leads to nonlocal elastic behavior.^[^ ^12^ ^]^ By the discussed analogy, in the static electric case, the resistor network shown in Figure 2b is expected to exhibit equivalent anomalous nonlocal behavior. In fact, the underlying equations are strictly equivalent mathematically. In Figure 2b, each electrical node is connected to the neighboring node by a resistor *R*_1_. In addition, each node is connected to its *N*‐th nearest neighbor by a resistor *R_N_*. In the example shown, we have chosen *N* = 2. This discrete resistor network is a model for the metawire structure shown in Figure 1c,d, where we also show the cases of *N* = 3 and *N* = 4. In addition, the depicted 1D design can easily be generalized to 2D and 3D lattices (*cf*. Figure S8 in Supporting Information).

**Figure 3:**
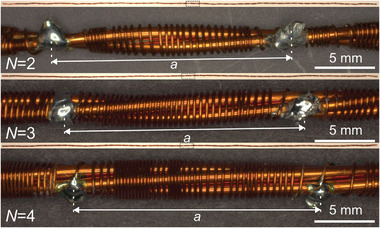
Photographs of manufactured samples following the design illustrated in Figure 1b–d for the three cases *N* = 2, 3, 4. The individual enameled copper wires are electrically connected at the soldering points separated by distance *a*, the metawire period, or lattice constant. The metawires have an overall length of ≈1 m.

**Figure** **3** exhibits photographs of three (*N* = 2, 3, 4) nonlocal metawires used in our experiments that follow the design blueprint shown in Figure 1b–d. They are composed of two types of enameled cylindrical copper wires with diameters *d*_1_ = 0.25 mm and *d_N_* = 1.1 mm, mediating the local connections and the nonlocal connections to the *N* ^th^‐nearest neighbor, respectively. The corresponding resistance ratios are *R*_1_/*R*_2_ = 100, *R*_1_/*R*_3_ = 100, and *R*_1_/*R*_4_ = 100. All samples have been handmade, starting by twisting the thicker copper wires and winding the thinner wires around. The wires have been hand‐soldered at the locations highlighted in green in Figure 1b–d, leading to a period or lattice constant of the metawire of about *a* = 2 cm. The three samples are composed of *M* = 52, 53, and 54 unit cells, respectively, leading to a total metawire length *Ma* of somewhat more than one meter.

To probe the effects of nonlocality, two different approaches are possible. (A) One can measure the resistance *R*(*L*) of a conducting wire (cf. Figure 1) versus the length *L* of the wire. This approach is connected to having to manufacture a large number of metawire samples with different lengths. (B) Alternatively, one can take a single fixed sufficiently long (or conceptually even infinitely long) metawire sample composed of *M* unit cells and measure the resistance *R*(*L*) between two points separated by distance *L* along the wire axis. For a local conductor, the two approaches deliver strictly identical results for the dependence *R*(*L*). For a nonlocal conductor, the two behaviors are still closely related, yet they are no longer strictly identical. To avoid the need for a very large number of metawire specimens, we follow approach (B).

**Figure 4:**
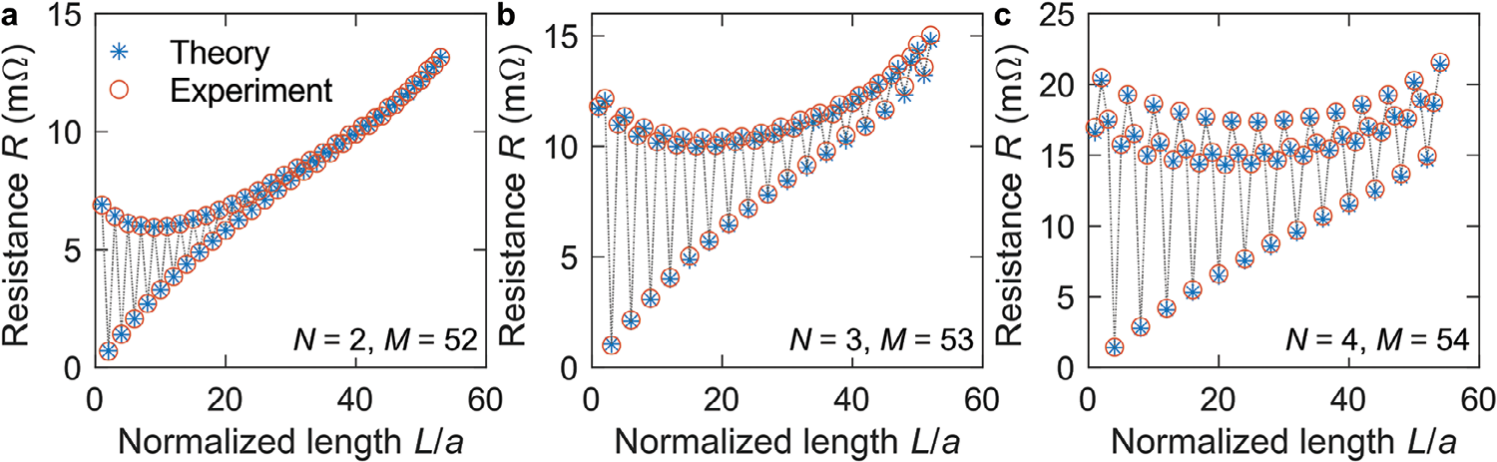
Effective metamaterial resistance *R*(*L*) versus normalized length *L*/*a* for the three cases *N* = 2, 3, 4 and *M* = 53, 52, 54. Experimental data are shown by the orange open dots, calculated results by the blue stars. The black straight lines are guides to the eye. We find pronounced oscillations of Ohm's resistance *R* versus *L*/*a* due to the nonlocality of the metawire. An ordinary local metal wire would exhibit a behavior following *R*(*L*)∝*L*/*a*.

The anticipated resistances are in the mΩ range, such that the influence of contact resistances in two‐point measurements is generally not negligible, which would lead to distortions of the experimental data. Therefore, we have rather performed four‐point measurements corresponding to the electrical resistance *R*(*L*) between two points separated by length or distance L=a,2a,…,50a. Experimental results are depicted in **Figure** **4**. The measured behavior *R*(*L*) is far from that of a local conductor according to *R*(*L*)∝*L*/*a*. We rather find pronounced oscillations of *R* versus *L*/*a* with period *N* for the three cases *N* = 2, 3, 4 also shown in Figure 3. These oscillations decay exponentially versus *L*/*a*. In other words, the response of the nonlocal metawire is highly sensitive to the distance between the two measurement positions. A small shift of one measurement point along the metawire, e.g., by just a single lattice constant *a*, can lead to a drastically different resistance, thereby significantly amplifying the possible resistance changes in local wires, potentially enabling improved remote electrical sensing.

Next, we explain the observed oscillation periods as well as the exponential decay lengths by comparison with the discrete resistor model shown in Figure 2b. We define ϕ_*n*_ as the electric potential at the node with integer index n=−∞,…−1,0,1,…,+∞ (cf. Figure 2b) and *Q_n_* as the charge of that node. In the static case, we have

(1) $$\frac{dQ_{n}}{dt}=0=+\left(\frac{\phi_{n}-\phi_{n-1}}{R_{1}}+\frac{\phi_{n}-\phi_{n-N}}{R_{N}}\right)-\left(\frac{\phi_{n+1}-\phi_{n}}{R_{1}}+\frac{\phi_{n+N}-\phi_{n}}{R_{N}}\right)$$

The four terms on the right‐hand side are the two current influxes (+) into and the two current outfluxes (−) from node *n*. Using the ansatz for the static Bloch eigenfunctions

(2) $$\phi_{n}=\tilde{\phi }\;\exp\left(ikan\right)$$

with prefactor $\tilde{\phi }$, we obtain the implicit equation for the spatial frequency (or “wavenumber”) *k* given by

(3) $$0=\left(1-\cos\left(ka\right)\right)+r_{N}\;\left(1-\cos\left(Nka\right)\right)$$

There are 2(*N* − 1)complex‐valued solutions k1,…,2N−1=Rek+iImk depend only on the dimensionless resistance ratio *r_N_* = *R*_1_ /*R_N_*. The real part of *k* determines the spatial oscillation period λ of the electrostatic potential via λ = 2π/|Re(*k*)|. The inverse of the imaginary part, *l* = 1/|Im(*k*)|, is the exponential decay length of this oscillation. In the absence of nonlocal connections, i.e., for *R_N_* → ∞⇒*r_N_* → 0, the only solution within the first Brillouin zone is *k* = 0, thus λ → ∞ and *l* → ∞. In the presence of nonlocal connections, e.g. for *r_N_* = *R*_1_ / *R_N_* = 100 and *N* = 2, 3, 4, we obtain λ ≈ 2*a*, 3*a*, 4*a* and *l* ≈ 10.0*a*, 17.3*a*, 28.3*a* by numerical solution. For the case of a bias voltage applied to a finite system instead of the left‐alone infinitely periodic system considered so far, the solution is that superposition of the 2(*N* − 1) Bloch solutions and the usual non‐Bloch solution ϕ_*n*_∝*an* (linear potential drop along the wire axis) that matches the boundary conditions. Therefore, the electric potential and observables such as the metawire resistance (cf. Figure 4a) oscillate versus length *L* with period *Na* for *N* = 2, 3, 4. Numerical solutions (see Methods) based on this lumped‐circuit model are shown in Figure 4 alongside the experimental data. The agreement is generally very good. We assign the remaining differences to fabrication imperfections of the handmade specimen (cf. Figure 3).

It is interesting to ask whether the experimental and theoretical findings shown in Figure 4a–c can be grasped by some sort of nonlocal effective‐medium description of electrical conduction, i.e., by a nonlocal generalization of a local version of Ohm's law. One should be aware that such effective‐medium descriptions are generally not unique.^[^ ^23^, ^24^ ^]^ We have pursued an approach based on higher‐order gradients, i.e., the current density at one location not only depends on the electric field at that same location but also on spatial derivatives of the electric field. Our corresponding results agree well with those shown in Figure 4a–c (see Note S2 in Supporting Information).

**Figure 5:**
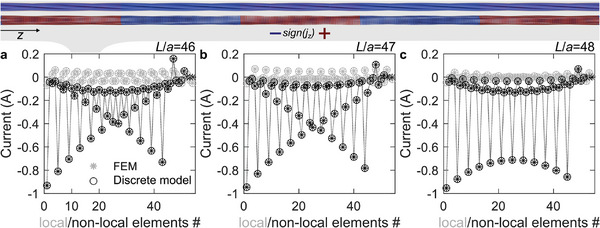
Illustration of calculated currents within the metawires. As an example, we chose *N* = 4, *M* = 54, and *L*/*a* = 46, 47, and 48 in panels a–c). The black data points refer to currents through the local connections, the gray data points to currents through the nonlocal connections. The stars refer to numerical finite‐element calculations, the open circles to the simple lumped‐resistor model (*cf*. Figure 2b). The straight lines are merely a guide to the eye. The false color representation at the top shows the current in 10 selected unit cells, separated into the local and the nonlocal contribution. As the left‐hand end of the metawire is grounded and a positive voltage is applied at a point on the right‐hand side, the overall electric current flows to the left (it is negative). Clearly, the current distribution exhibits a complex oscillation pattern. In some parts of the metawire, the current flows into the opposite, i.e., positive, direction.

On the basis of our above analytical discussion based on describing the metawires in Figure 1b–d as a network of lumped resistors, the local electric currents can be negative with respect to the overall positive current running through the metawire. Also, the spatial current distribution is expected to depend on the boundary conditions. To further test this interpretation and the theory results shown in Figure 4, we have performed numerical finite‐element numerical calculations for the three metawire geometries depicted and defined in Figure 1b–d. In these calculations, we assume a constant scalar electric conductivity σ_0_ for all metal parts (zero else wise) and solve the equation ***j*** (***r***) = σ_0_ ***E*** (***r***) in three dimensions under the boundary conditions imposed by the applied bias voltage. Details are described in Methods. **Figure** **5** shows examples of calculated currents for the cases *N* = 4, *M* = 54, and *L*/*a* = 46, 47, and 48 in panels (a), (b), and (c). To illustrate the complex behavior, we separately depict the current in the local wires and in the nonlocal wires, respectively. The results of these continuum‐model calculations are compared with the results of the lumped‐resistor model. At the top, we illustrate the current distribution on the wire surfaces by a false‐color scale superimposed onto the model (cf. Figure 1d). Red color corresponds to a current in the direction of the applied electric field, blue color to a current in the opposite direction, and green color to a near‐zero current. For clarity, we separately depict the current in the local wires and in the nonlocal wires, respectively. As expected on the basis of our above analytical calculations based on the static Bloch eigenfunctions, we find an oscillatory behavior of the current with spatial period λ = *Na* = 4*a*, leading to alternating positive and negative currents. We also find that the “standing‐wave” behavior depends on the boundary conditions, which are different for the two cases *L*/*a* = 47 and 48 shown as examples. The found static (zero frequency) negative charge currents can be seen as analogous to backward energy currents or flows in, e.g., elastic metamaterials exhibiting backward waves at finite frequencies.^[^ ^25^, ^26^, ^27^, ^28^ ^]^

## 3 Conclusion

In ordinary metals and conductors at optical frequencies, nonlocal effects of the conductivity can play a role on the atomic scale, i.e., on the scale of about one or just a few lattice constants. Herein, we have introduced metallic metawires that, in the static or dc case, exhibit pronounced effects of nonlocal conductivity on the scale of ten or more metawire periods. Conceptually, the characteristic length can even be made to diverge by design. As a result, effects of nonlocality become extremely prominent and manifest as pronounced oscillations of the resistance of the metawire versus the length of the metawire. Some contributions to the total current point in the backward direction. This behavior is related to backward waves. Such sensitive dependence of the resistance on the boundary conditions might find applications in electrical sensing.

## 4 Experimental Section

### Numerical Solutions

For the metawire calculation based on the lumped‐resistor model, the method delineated in Wu^[^ ^29^ ^]^ and Izmailianet et al.^[^ ^30^ ^]^ was utilized. This approach entails constructing the Laplace/Kirchhoff matrix, denoted as ***L***, for a resistance network consisting of *M* nodes connected by resistors, with a resistivity, *r*_αβ_ or, alternatively, with a conductivity represented as *c*_αβ_ = 1/*r*_αβ_, between nodes α and β. In the equation ***I*** = ***L U***, where ***I*** and ***U*** denote the current and voltage at each node, respectively,

(4) $$L=\left(\begin{array}{ccccc} c_{1} & -c_{12} & -c_{13} & \cdots & -c_{1n}\\ -c_{21} & c_{2} & -c_{23} & \cdots & -c_{2n}\\ -c_{31} & -c_{32} & c_{3} & \cdots & -c_{3n}\\ \vdots & \vdots & \vdots & \ddots & \vdots \\ -c_{n1} & -c_{n2} & -c_{n3} & \cdots & c_{n} \end{array}\right)$$

with $c_{i}=\sum_{\begin{array}{c} j=1\\ j\neq i \end{array}}^{M}c_{ij}$. ***L*** has eigenvectors, denoted as ***v**_j_* = (*v*_*j*1_,*v*_*j*2_, ⋅⋅⋅, *v_jM_*), for *j* = (1, 2, ⋅⋅⋅, *M*), and corresponding eigenvalues λ_*j*_. From these eigenvectors and eigenvalues, it has been proven in^[^ ^29^ ^]^ that the resistance *R*_αβ_ between any two arbitrary points α and β in the resistor network can be calculated following

(5) $$R_{\alpha \beta }=\sum_{j=2}^{M}\frac{{\left|v_{j\alpha }-v_{j\beta }\right|}^{2}}{\lambda_{j}}$$

This method can be easily extended to accommodate different types of boundary conditions, such as periodic boundaries or in the case of an infinite metawire. For more details, the reader to the Supporting Information was reffered.

To compute the current of the *n*‐th element, It was recognized that the first equation is a redundant equation of the Laplacian. By disregarding the first row and column of ***L***, a matrix ***H***, which was a (*M* − 1) × (*M* − 1) matrix was defined. The inverse of ***H*** was well defined, allowing to express the equation as ***U*** = ***H*** ^−1^ ***I***. The resistance of the *n*‐th element was known. Therefore, for a given imposed current, calculating the voltage between the two nodes to which the element was connected, immediately allows for calculating the current passing through each element.

### Continuum‐Model Calculations

The resistances of the finite‐size metawire for all cases (*N* = 2, 3, and 4) were calculated by solving Ohm's equation:

(6) j=σE

where ***j*** represents the current density, σ denotes the conductance, and ***E*** stands for the electric field.

This equation was solved using the AC/DC module in the commercial software COMSOL Multiphysics, employing its MUMPS solver. A constant current at the boundary of the *n*‐th welding point was applied, with a total current of 1 A. Additionally, the ground boundary condition was imposed at the starting node of the metawire. The material properties used for all calculations where σ_copper_ = 5.998 × 10^7^ S/m for the wires and σ_solder_ = 6.67 × 10^6^ S/m for the solder at the welding points.

For the calculation of the current, *I*, in some *n*‐th elements, the surface integral of the current density across the cross‐sectional area *A* of the wires at the middle of the unit cell was computed.

(7) $$I=\int \;\;\;\int_{A}j\cdot dA$$

### Resistance Measurements

To measure the resistance between two nodes of the metawire, the four‐wire connection method was employed. This method was implemented with the help of the precision source/measurement unit Keysight B2900 series, combined with Kelvin probes. The Kelvin probes were used to connect the low‐sense and force wire to one node, and the high‐force and sense wires to the other. A constant current of *I* = 1 A and measured the voltage, *U*, between these probes was applied. Finally, the resistance via *R* = *U*/*I* was determined.

## Conflict of Interest

The authors declare that they have no conflict of interest.

## Supporting information



Supporting Information

## Data Availability

The data that support the findings of this study are openly available in [RADAR4KIT] at [https://doi.org/10.35097/kAmkUzRyKMMjMhbg], reference number [0].
