# Supplementary material for: Nonlocal Conduction in a Metawire
Source: Adv Mater. 2025 Feb 21;37(13):2415278. doi: 10.1002/adma.202415278 (PMC11962673; doi:10.1002/adma.202415278)
Supplement: Supplementary file 1 — Supporting Information [file ADMA-37-2415278-s001.pdf]

ADVANCED MATERIALS

Supporting Information

for *Adv. Mater.*, DOI 10.1002/adma.202415278

# Nonlocal Conduction in a Metawire

*Julio Andrés Iglesias Martínez*, Yi Chen*, Ke Wang and Martin Wegener**

Supporting Information

# Nonlocal Conduction in a Metawire

*Julio Andrés Iglesias Martínez*, Yi Chen*, Ke Wang, and Martin Wegener**

J.A. Iglesias Martínez, Y. Chen, and M. Wegener
Institute of Nanotechnology, Karlsruhe Institute of Technology (KIT), Karlsruhe 76128, Germany.
E-mail: julio.martinez@kit.edu; yi.chen@partner.kit.edu; martin.wegener@kit.edu.

J.A. Iglesias Martínez, Y. Chen, Ke Wang, and M. Wegener
Institute of Applied Physics, Karlsruhe Institute of Technology (KIT), Karlsruhe 76128, Germany.

This file includes:

Supplemental Figures S1 – S8

Supplemental Notes 1 – 2

Supplemental References: 1 – 3

**Supplemental Note 1. Resistance of lumped-circuit model**

As discussed in the main paper, we follow the method described by Wu [1] and Izmailianet et al. [2]. We consider a resistance network with $M$ nodes with resistors connecting the nodes and a conductivity $c_{\alpha\beta} = 1/r_{\alpha\beta}$ between the nodes $\alpha$ and $\beta$. Using the two Kirchhoff laws, one arrives at the Laplacian matrix, $\boldsymbol{L}$, given by

$$\boldsymbol{L} = \begin{pmatrix} c_1 & -c_{12} & -c_{13} & \cdots & -c_{1n} \\ -c_{21} & c_2 & -c_{23} & \cdots & -c_{2n} \\ -c_{31} & -c_{32} & c_3 & \cdots & -c_{3n} \\ \vdots & \vdots & \vdots & \ddots & \vdots \\ -c_{n1} & -c_{n2} & -c_{n3} & \cdots & c_n \end{pmatrix}.$$

With $c_i = \sum_{\substack{j=1 \\ j\neq i}}^{M} c_{ij}$, the eigenvectors $v_j$, and the eigenvalues $\lambda_j$, it is possible to calculate de resistance between the nodes $\alpha$ and $\beta$ following

$$R_{\alpha\beta} = \sum_{j=2}^{M} \frac{\left|v_{j\alpha} - v_{j\beta}\right|^2}{\lambda_j}.$$

For periodic boundary conditions, such as in the example shown for $N = 2$ and $M = 8$ in Figure S1, the resulting Laplacian matrix is circulant. Therefore, the eigenvectors $v_j$, and eigenvalues $\lambda_j$ are known. With $\psi = \exp\left(\frac{2\pi i}{M}\right)$, they are given by

$$v_j = \frac{1}{\sqrt{n}}\left(1, \psi^j, \psi^{2j}, \cdots, \psi^{(M-1)j}\right)$$

$$\lambda_j = c_0 + c_1\psi^j, c_2\psi^{2j} + \cdots + c_{(n-1)}\psi^{(M-1)j}$$

For $n$ nodes, with conductances $c_1$ and $c_N$, the resistance between the nodes at positions $x_\alpha$ and $x_\beta$ of a metawire with lattice constant $a$ is:

$$R_{\alpha\beta} = \frac{1}{M}\sum_{j=1}^{M-1} \frac{1 - \cos\left(2\frac{\left(x_\alpha - x_\beta\right)}{a} j \frac{\pi}{M}\right)}{c_1\left(1 - \cos\left(2\, j\frac{\pi}{M}\right)\right) + c_N\left(1 - \cos\left(2\, Nj\frac{\pi}{M}\right)\right)} \tag{1}$$

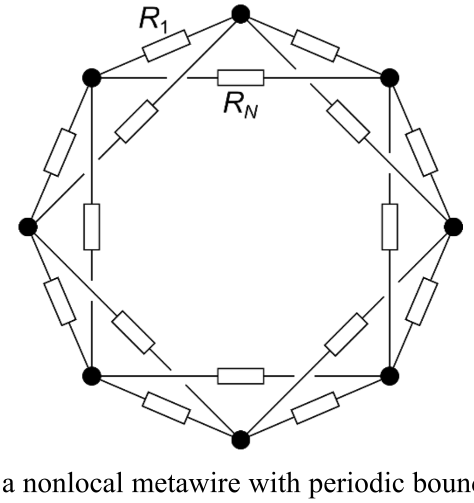


**Figure S1.** Discrete model for a nonlocal metawire with periodic boundary conditions for the example of $N = 2$ and $M = 8$.

As an illustration, the resistance as a function of the normalized length $L/a$ for $N=2,3$ and $4$, $R_1/R_N = 100$ and for $M=50$ is depicted in Figure S2. We find oscillations with period $Na$.

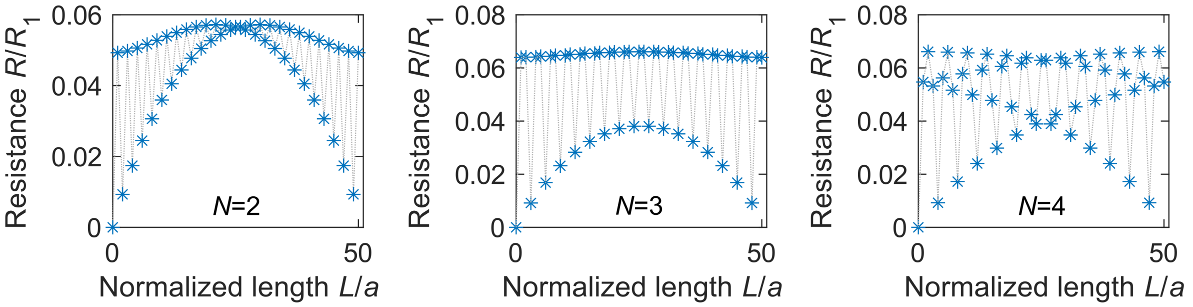


**Figure S2.** Normalized effective metawire resistance $R/R_1$ versus normalized length $L/a$ for the three cases $N=2,3,4$ and $M=50$, with periodic boundary conditons. We again find pronounced oscillations of Ohm's resistance versus $L/a$ due to the nonlocality of the metawire with periodicity of $Na$. An ordinary local metal wire would exhibit a behavior following $R(L) \propto L/a$.

We can use Eq. (1) to study the limit of $M \to \infty$ to obtain the infinite metawire resistance $R(l)$ for an arbitrary normalized distance, $l = \frac{|x_\alpha - x_b|}{a}$,

$$R(l) = \lim_{M\to\infty} R_{\alpha\beta} = \frac{1}{\pi}\int_0^\pi \frac{1-\cos(l\,\theta)}{c_1(1-\cos(\theta)) + c_N(1-\cos(N\,\theta))} \mathrm{d}\theta.$$

As for the case of periodic boundary conditions, we find oscillations. Figure S3 depicts examples, evidencing that the boundary conditions do not change the qualitative behavior.

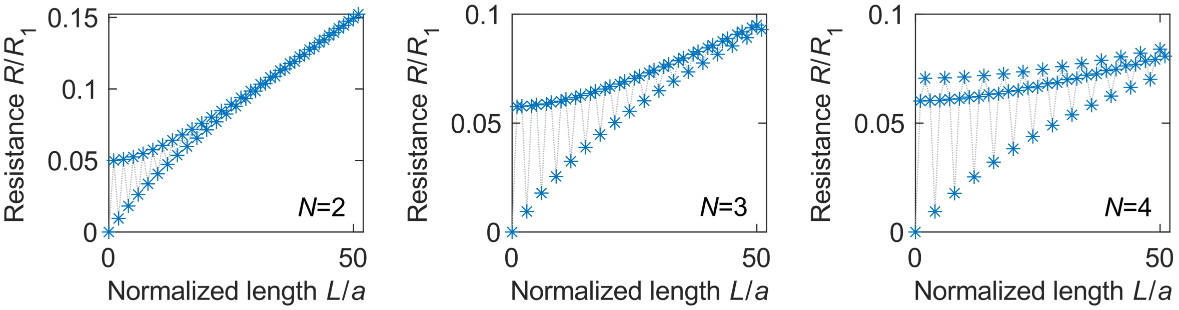


**Figure S3**. Normalized effective metawire resistance $R/R_1$ versus normalized length $L/a$ for the three cases $N=2,3,4$ and $M\to\infty$. We also find pronounced oscillations of Ohm's resistance versus $L/a$ due to the nonlocality of the metawire with periodicity of $Na$. An ordinary local metal wire would exhibit a behavior following $R/R_1 \propto L/a$.

As mentioned in the main paper, we have also investigated the case of not only having local interactions and one type of nonlocal interactions, but rather also of having local interaction and several different nonlocal interactions combined. As an example, we consider $N = 1$ and $N = 2$ and $N = 3$ and $N = 4$ combined with the resistance ratios $r_2 = R_1/R_2$, $r_3 = R_1/R_3$, and $r_4 = R_1/R_4$. For $r_2 = r_3 = r_4 = 100$, as depicted at Figure S4, where the effective resistance $R(L)$ versus normalized length $L/a$ is shown for $M = 50$, we find hardly any oscillations. Minor nonlocal effects are visible close to the boundaries. This absence of oscillations can be understood when inspecting the corresponding decay lengths. Here, the Bloch eigensolutions are connected to $k \approx \{(3.14 \pm 0.51\mathrm{i}), \pm(1.73 \pm 0.59\mathrm{i}),\}/a$, corresponding to a wavelength and decay length of $\lambda \approx \{2.00, 3.62\}a$ and $l \approx \{1.97, 1.89\}a$, respectively.

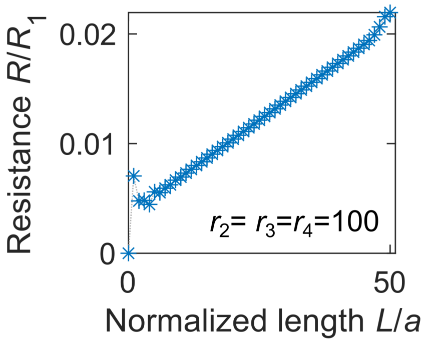


**Figure S4.** Normalized effective metawire resistance $R/R_1$ versus normalized length $L/a$ for $r_2 = r_3 = r_4 = 100$ and $M = 50$. For this case, we do not find pronounced oscillations of Ohm's resistance versus $L/a$. The only remarkable nonlocal effect is a jump near the left boundary.

We have also studied all possible combinations for the local interactions being finite, and only one of the three nonlocal interactions being zero. For example, $r_3 = 0$ is equivalent to the absence of third order interactions via $R_3 \to \infty$. Three cases exist, namely first $r_2 = r_3 = 100$ and $r_4 = 0$, second $r_2 = r_4 = 100$ and $r_3 = 0$, and third $r_3 = r_4 = 100$ and $r_2 = 0$. Fig. 5 exhibits the resistance as function of the normalized length $L/a$ for $M = 50$ for these three cases. For the first and third case, we find oscillations that are rapidly damped, again due to a small decay length. In contrast, for the second case, pronounced oscillations appear with a periodicity of $2a$.

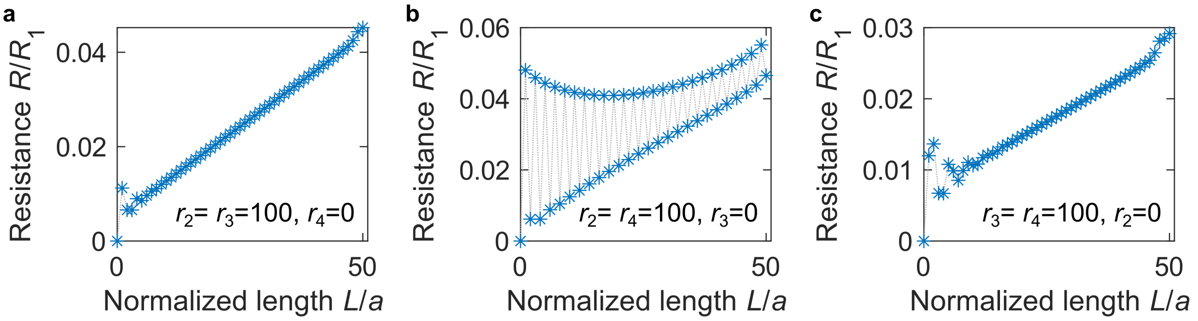


**Figure S5**. Normalized effective metawire resistance $R/R_1$ versus normalized length $L/a$ for the three combinations (all with $M = 50$): (a) $r_2 = r_3 = 100$ and $r_4 = 0$, (b) $r_2 = r_4 = 100$ and $r_3 = 0$, and (c) $r_3 = r_4 = 100$ and $r_4 = 0$. We only find pronounced oscillations of Ohm's resistance versus $L/a$ for panel b, where the periodicity is $2a$. For panels (a) and (c), the oscillations are rapidly damped and the only nonlocal effect are anomalies close to the boundaries.

To intuitively understand this behavior, let us consider the absence of the local interactions. In this approximation, the case with second and fourth-order nonlocal interactions ($N = 2$ and $N = 4$) is equivalent to two disconnected metawires with nonlocal interactions to the second neighbor, leading to a periodicity of $2a$. Nevertheless, since $R_2/R_4 = 1$ the nonlocal effects are expected to be small. Subsequently, if we switch the local interaction back on, it is possible to approximate the metawire by a single nonlocal interaction of $N = 2$, as shown in Fig. 4a of the main text, yet with a different effective value for $r_2$. This rule can be generalized: If the resistances of the nonlocal interactions are comparable, we obtain pronounced oscillations if the nonlocal interactions have a greatest common divisor different from unity.

We have also calculated the static Bloch eigensolutions with their corresponding decay lengths and wavelengths:

| | |
|---|---|
| $r_2 = r_3 = 100$ and $r_4 = 0$ | $k \approx \{\pm(2.28 \pm 0.55\mathrm{i})\}/a$<br>$\lambda \approx 2.76a,\ l \approx\ 1.83\ a$ |

$r_2 = r_4 = 100$ and $r_3 = 0$     $k \approx \{(3.14 \pm 0.04\mathrm{i}), \pm(1.57 \pm 0.48\mathrm{i})\}/a$ ,
$\lambda \approx \{2.00, 4.00\}a,\ l \approx \{22.38, 2.07\}a$

$r_3 = r_4 = 100$ and $r_2 = 0$     $k \approx \{(3.14 \pm 0.56\mathrm{i}), \pm(1.73 \pm 0.28\mathrm{i}),\}/a$ ,
$\lambda \approx \{2.00, 3.62\}a,\ l \approx \{1.77, 3.53\}a$

We find that all decay lengths $l$ are small, except for the mode corresponding for $r_2 = r_4 = 100$ and $r_3 = 0$ (second case). Here $\lambda \approx\ 2\ a$ and $l \approx 22\ a$ – confirming our above interpretation.

**Supplemental Note 2. Effective-medium model**

We start from a 1D lumped-circuit model for the nonlocal metawire ($N = 2$) with lattice constant $a$, as shown in Fig. 6. The electric potential at node $n$ is labeled as $\phi_n$. The electric current from the left to the right through the cross section (dashed vertical straight lines) at the node $n$ of the model is

$$I_n = \frac{1}{2}\left(\frac{\phi_{n-1} - \phi_n}{R_1} + \frac{\phi_{n-2} - \phi_n + \phi_{n-1} - \phi_{n+1}}{R_2}\right) + \frac{1}{2}\left(\frac{\phi_n - \phi_{n+1}}{R_1} + \frac{\phi_{n-1} - \phi_{n+1} + \phi_n - \phi_{n+2}}{R_2}\right)$$

To obtain a continuum version of this equation, we consider the following Taylor expansion of the node potential

$$\phi_{n+M} = \phi_n + \sum_{\alpha=1}^{\infty} \frac{1}{\alpha!}\left(\frac{\mathrm{d}^\alpha \phi(x)}{\mathrm{d}x^\alpha}\right)(Ma)^\alpha.$$

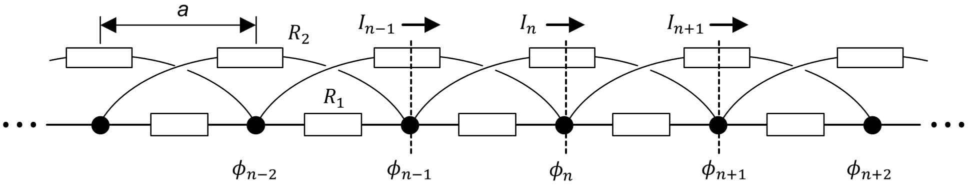


**Figure S6.** Lumped-circuit model of a 1D metawire with second-order nonlocal interaction (i.e., $N = 2$).

By substituting the above expansion into the formula of the electric current flow, we obtain

$$I^{\mathrm{eff}}(x) = C_1 E^{\mathrm{eff}}(x) + C_3 \frac{\mathrm{d}^2 E^{\mathrm{eff}}(x)}{\mathrm{d}x^2} + C_5 \frac{\mathrm{d}^4 E^{\mathrm{eff}}(x)}{\mathrm{d}x^4},$$

with $E^{\mathrm{eff}}(x) = -\mathrm{d}\phi(x)/\mathrm{d}x$ representing the effective-medium electric field and the coefficients being $C_1 = 4a/R_2 + a/R_1$, $C_3 = 5a^3/(3R_2) + a^3/(6R_1)$, and $C_5 = 17a^5/(60R_2) + a^5/(120R_1)$. We have truncated the expansion at the fourth order. We see that the effective-medium current not only depends on the electric field itself, but also depends on gradients of the electric field. These gradients represent the nonlocality [3]. Odd-order gradients are zero because of reciprocity.

From the continuity of $I_n$ for the lumped-circuit model, we obtain the continuity law for the continuum model

$$\frac{\mathrm{d}I^{\mathrm{eff}}(x)}{\mathrm{d}x} = -C_1 \frac{\mathrm{d}\phi^2(x)}{\mathrm{d}x^2} - C_3 \frac{\mathrm{d}^4\phi(x)}{\mathrm{d}x^4} - C_5 \frac{\mathrm{d}^6\phi(x)}{\mathrm{d}x^6} = 0.$$

To obtain the resistance of a metawire with total length $L$ from the effective-medium model, we assign the left and right boundaries with the potentials $\phi(0) = 0$ and $\phi(L) = \phi_{\max}$, respectively. Furthermore, we have four additional boundary conditions for the above sixth-order differential equation

$$\frac{\mathrm{d}\phi(0)}{\mathrm{d}x} + \frac{\mathrm{d}\phi(L)}{\mathrm{d}x} = 0, \quad \frac{\mathrm{d}\phi^2(0)}{\mathrm{d}x^2} + \frac{\mathrm{d}\phi^2(L)}{\mathrm{d}x^2} = 0, \quad \frac{\mathrm{d}\phi^3(0)}{\mathrm{d}x^3} + \frac{\mathrm{d}\phi^3(L)}{\mathrm{d}x^3} = 0, \quad \frac{\mathrm{d}\phi^4(0)}{\mathrm{d}x^4} + \frac{\mathrm{d}\phi^4(L)}{\mathrm{d}x^4} = 0.$$

After solving for the electric potential $\phi(x)$, the effective Ohmic resistance is then calculated as

$$R^{\mathrm{eff}}(L) = \frac{\phi(0) - \phi(L)}{I^{\mathrm{eff}}}.$$

In the calculation for $N = 2$ and $R_1/R_N = 100$ , we have chosen the effective-medium parameters $C_1 = 4a/R_N$, $C_3 = 0.81a^3/R_N$, $C_5 = 0.04a^5/R_N$ to obtain reasonable agreement between the effective-medium and the lumped-circuit model concerning the oscillation period and the oscillation decay length. The overall behavior is well reproduced by the effective-medium theory (see Fig. 7a). These results for $N = 2$ can easily be generalized to the other two cases discussed in the main paper, i.e., for $N = 3$ (see Fig. 7b) and $N = 4$ (see Fig. 7c). Again, we obtain good qualitative agreement. Herein, we have used the following effective-medium parameters: $C_1 = 4.5a/R_N$, $C_3 = 2.0512a^3/R_N$, and $C_5 = 0.2342a^5/R_N$ for $N = 3$ and $C_1 = 8.0a/R_N$, $C_3 = 6.4839a^3/R_N$, and $C_5 = 1.3156a^5/R_N$ for $N = 4$.

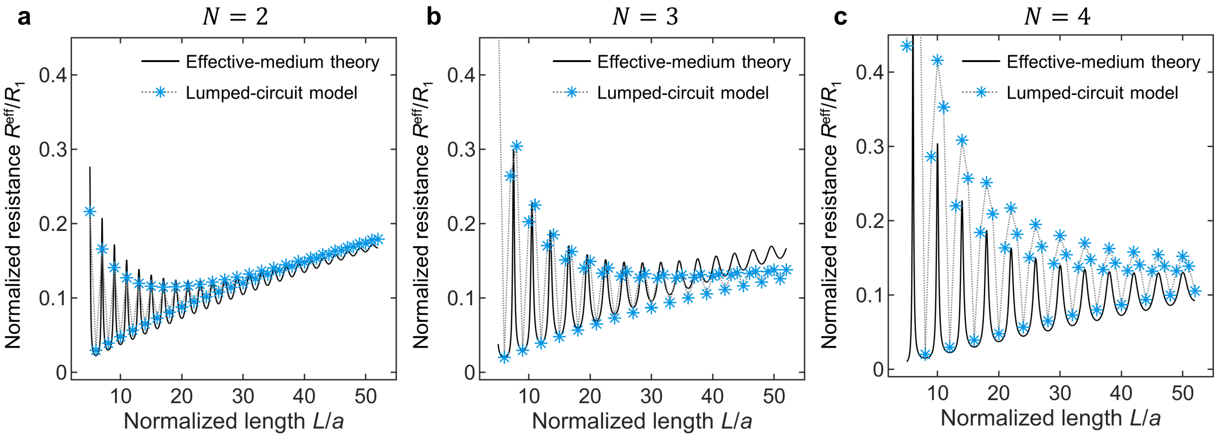


**Figure S7.** Calculated electric resistance versus wire length from the lumped-circuit model and the effective-medium theory (solid black curves). (a) $N = 2$, (b) $N = 3$, and (c) $N = 4$.

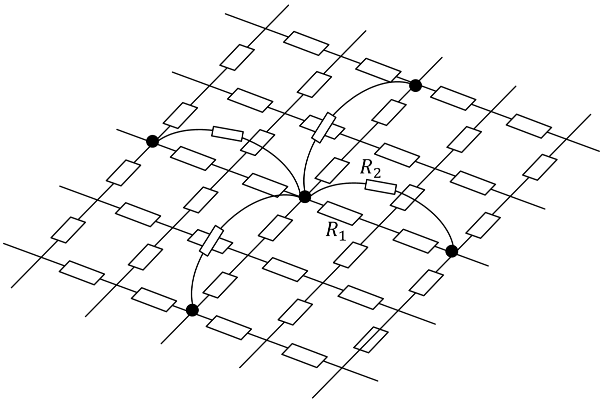


**Figure S8.** Scheme of a discrete model for a 2D nonlocal metamaterial (or metawire material) with second-order ($N = 2$) nonlocal interactions along two orthogonal directions. For clarity, only the nonlocal connections emerging from the central black node are shown. It is likewise straightforward to build a 2D square lattice of metawires following Fig. 2b-d of the main paper or a 3D simple-cubic lattice.

**References:**

[1] F. Wu, Theory of resistor networks: the two-point resistance. J. Phys. A Math. Gen. **37**, 6653 (2004).

[2] N. S. Izmailian, R. Kenna, and F. Y. Wu, The two-point resistance of a resistor network: a new formulation and application to the cobweb network. J. Phys. A Math. Gen. **47**, 35003 (2013).

[3] L. D. Landau, J. S. Bell, M. J. Kearsley et al., *Electrodynamics of continuous media*, Vol. 8, Elsevier, Amsterdam, The Netherlands **2013**.
